# Clinical characteristics of *infants* hospitalized with early congenital syphilitic nephropathy: a single-center retrospective cross-sectional study in China

**DOI:** 10.1186/s12887-023-04250-4

**Published:** 2023-09-04

**Authors:** Caiying Wang, Wenhui Lun, Lin Pang

**Affiliations:** 1 Department of Paediatrics, Beijing Ditan Hospital, Capital Medical University, Jingshun East Street, No. 8, Beijing, 100015 China; 2 Department of Dermatology, Beijing Ditan Hospital, Capital Medical University, Beijing, China

**Keywords:** Early congenital syphilis, Syphilitic nephropathy, Clinical characteristics

## Abstract

**Background:**

Early studies claimed that early congenital syphilitic (CS) nephropathy was rare, and systematic studies about this disease are absent, which may lead to poor awareness of early CS nephropathy in clinicians and result in misdiagnosis and poor patient prognosis. The present study systematically and comprehensively *analyzes* the clinical characteristics of *infants* with early CS nephropathy hospitalized in Beijing Ditan Hospital, an infectious disease hospital in China in order to improve the understanding and management of this disorder.

**Methods:**

Data of the children with early CS from July 1, 2008, to December 31, 2021, were collected from the electronic medical record system of the hospital. Each patient’s demographic characteristics, clinical history, mother’s history of syphilitic infection, and laboratory values were extracted. The patients were enrolled to either the nephropathy group or the non-nephropathy group depending on diagnosis. Descriptive statistics was used to report basic demographics, clinical and laboratory test values, and variables were compared between the two groups by nonparametric tests, t test or *χ*^2^ tests.

**Results:**

Of the 122 children with early CS enrolled, 24(19.7%) were diagnosed with early CS nephropathy. All of the children with CS nephropathy were young infants < 6 months old. A majority of them showed typical congenital syphilitic skin lesions, but a quarter of them did not have skin lesions. Compared with non-nephropathic children with early CS, those with nephropathy had higher frequency of hepatosplenomegaly, fever, edema, gastrointestinal (GI) symptoms, and anemia, as well as decreased C3 levels. Urinalysis results showed hematuria in all patients with early CS nephropathy, with proteinuria and renal function impairment in 91.7% and 12.5% of the patients, respectively. Nephritic-type nephrotic syndrome and glomerulonephritis were diagnosed in 45.8% and 54.2% of these patients, respectively. All *infants* with CS nephropathy were cured or improved after appropriate treatments.

**Conclusion:**

*Infants with* early CS nephropathy often presented with nephritic-type nephrotic syndrome or glomerulonephritis, and the typical skin lesions, fever, hepatosplenomegaly, and *edema, etc.*, were its common clinical presentations, and these characteristics could help with the diagnosis. But for *infants with nephropathy* who did not have typical clinical presentations, CS should also be screened as an important etiology to avoid misdiagnosis.

**Supplementary Information:**

The online version contains supplementary material available at 10.1186/s12887-023-04250-4.

## Background

Despite recent advances in treatment to eliminate mother-to-child transmission of syphilis, congenital syphilis (CS) is still a public-health concern in both developed and developing countries [1–3]. This might be due to the increasing prevalence of syphilis among women of childbearing age and inadequate awareness of the disease in some pregnant women [2, 4−6. Therefore, improving clinicians’ understanding and management of CS is important.

Early CS, defined as CS in children aged < 2 years, can seriously affect multiple systems in children and may even be fatal. Its clinical characteristics can be complex and diverse, and it is important to recognize all the clinical features of CS and begin treatment as soon as possible. *CS nephropathy was rarely encountered in the literature* [7], and only some case reports have been published on early CS nephropathy, and most affected patients presented as nephrotic syndrome [8–18], but systematic studies are absent. This has led to poor awareness of early CS nephropathy in clinicians, resulting in misdiagnosis, missed diagnosis, and poor patient prognosis. Therefore, a systematic study to comprehensively evaluate early CS nephropathy to improve clinicians’ knowledge and management of this disease is needed.

In the present study, we collected the clinical information of children with early CS nephropathy hospitalized in our hospital, and analyzed its prevalence and clinical characteristics, laboratory test results, treatments, and prognosis.

## Methods

### Study design and participants

This was a retrospective cross-sectional study in children with early CS who were admitted to Beijing Ditan Hospital, Capital Medical University, Beijing, China, from July 1, 2008, to December 31, 2021. The study protocol was approved by the hospital ethics committee.

Inclusion criteria were children diagnosed as having early CS (the diagnostic criteria are shown in S1). Children who were treated with penicillin, third-generation cephalosporins, or macrolides for > 7 days at other hospitals were excluded.

### Data collection and group assignments

We reviewed the children’s medical records in the electronic medical record system of the hospital and collected data on their demographics, clinical characteristics, laboratory tests, imaging reports, treatments, and follow-up outcomes. If a patient underwent multiple clinic visits and tests, the patient’s pre-treatment test results that were used for analysis were the results of the first visit and first test. We assigned the enrolled children to either the nephropathy group or the non-nephropathy group depending on diagnosis.

Diagnostic criteria for early CS were based on the relevant guidelines issued by the Chinese Center for Disease Control and Prevention [19]. Diagnostic criteria for CS nephropathy (S2) were based on a previously published study [11]. Diagnostic criteria for nephrotic syndrome (S3), nephritic-type nephrotic syndrome (S4), and glomerulonephritis were based on the relevant Chinese guidelines [20]. Syphilis treatments followed the Zhufutang Practical Pediatrics [21] and World Health Organization (WHO) Guidelines [22] (S6). All these criteria and guidelines were described in the Supplementary Materials.

### Statistical analysis

Data were presented as medians with interquartile ranges (IQRs) or percentages with 95% confidence limits for continuous or categorical data, respectively. We compared the two groups using nonparametric tests and *t* test for non-normally distributed continuous data and normally distributed continuous data, respectively; and *χ*^2^ tests for categorical data. A *P* < 0.05 was considered statistically significant. All analyses were performed using SPSS version 22.0 (IBM Corp., Armonk, New York, USA).

### Patient and public involvement

*There was no patient or public involvement in this study.*

## Results

### Baseline characteristics

A total of 122 children with early CS were included in this study. Twenty-eight had nephropathy, and of these, five also had cytomegalovirus (CMV) infection. One of the five children had complete remission of nephropathy after syphilis *rather than* antiviral CMV treatment, confirming the diagnosis of CS nephropathy. The remaining four were treated with ganciclovir antiviral therapy and penicillin for syphilis simultaneously. Therefore, we excluded these four children in accordance with diagnostic criteria for CS nephropathy. The remaining 24 children (24/122, 19.7%) met the diagnostic criteria. All children with CS nephropathy were young infants < 6 months old (median age, 1.6 months). Except for one abandoned infant whose mother’s medical history was unknown, all the *infants’* mothers had clear histories of syphilitic infection during pregnancy and did not receive standard syphilis treatment to interrupt mother-to-child transmission (S7).

In children with early CS, there were no statistical differences in demographic characteristics between those with and those without nephropathy (*P* > 0.05).

### Clinical presentations

**Fig. 1:**
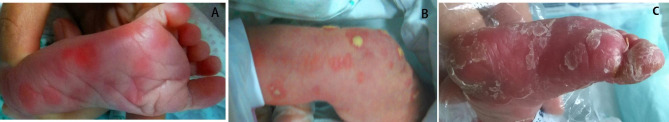
The rash of congenital syphilis includes macular, copper-colored rashes on the palms and soles, or other body parts **(A)**, vesiculobullous and pustules eruptions **(B)** and desquamation of feet and palm **(C)**

The clinical presentations of *infants* with early CS nephropathy varied significantly (Table 1). Typical syphilitic skin lesions (Fig. 1) presented in 75.0% (18/24) of *infants*, while 25% (6/24) of the patients showed no typical syphilitic skin lesions. Hepatosplenomegaly, fever, edema and gastrointestinal (GI) symptoms such as vomiting and abdominal distention were also common. Other clinical presentations included pale skin, petechia, nasal congestion, jaundice, and pseudoparalysis.

In the clinic, a higher proportion of patients with CS nephropathy had hepatosplenomegaly, fever, edema, and GI symptoms than those with early CS but no nephropathy (*P* < 0.05). For other clinical symptoms, differences between the two groups were not statistically significant.

**Table 1:** Comparisons of clinical presentations between children with congenital syphilis with or without nephropathy

Clinical presentations (N, %)	With nephropathy (N = 24) (cases, percentage, 95% CI of percentage)	Without nephropathy (N = 94) (cases, percentage, 95% CI of percentage)
Skin lesions (rash or flaking)	18 (75.0%, 56.3-93.7%)	75 (79.8%, 71.5-88.1%)
Hepatosplenomegaly*	17 (70.8%, 51.2-90.4%)	45 (47.9%, 37.6-58.2%)
Fever*	15 (62.5%,41.6-83.4%)	32 (34.0%, 24.3-43.8%)
Edema*	14 (58.3%, 37.1-79.6%)	4 (4.3%, 0.1-8.4%)
Vomiting, bloating, or diarrhea*	14 (58.3%, 37.1-79.6%)	19 (20.2%,11.9-28.5%)
Pale skin	9 (37.5%, 16.6-58.4%)	20 (21.3%, 12.8-29.7%)
Stuffy nose	8 (33.3%,13.0-53.7%)	19 (*20.2%*, 11.9-28.5%)
Cough	8 (33.3%,13.0-53.7%)	18 (19.1%, 11.0-27.3%)
*Long bone radiographic abnormalities consistent with osseous syphilis (N, %)* ^*#*^	*7/22 (31.8%, 10.7-53.0%)* *(checked in 22 children)*	*37/92 (40.2%, 30.0-50.4%)* *(checked in 92 children)*
Jaundice	5 (20.8%, 3.3-38.4%)	21 (22.3%, 13.8-30.9%)
Petechiae	3 (12.5%, 2.7-32.4%)	4 (4.3%, 1.2-10.5%)
Dark urine	2 (8.3%, 1.0-27.0%)	0 (0%, 0-3.8%)
Pseudoparalysis	1 (4.2%, 0.1-21.1%)	6 (6.4%, 1.3-11.4%)
Convulsions	1 (4.2%, 0.1-21.1%)	2 (2.1%, 0.3-7.5%)
No obvious symptoms or signs	1 (4.2%, 0.1-21.1%)	2 (2.1%, 0.3-7.5%)

*P < 0.05

# Long bone radiographic abnormalities consistent with osseous syphilis included metaphyseal osteochondritis, metaphyseal or diaphyseal osteitis, periosteal reaction, or even pathologic fracture

### Laboratory and imaging tests

As shown in Table 2, all *infants* (24/24,100%) with CS nephropathy had hematuria, with a median microscopic red blood cell (RBC) count of 46.7/high-power field (HPF). Proteinuria was reported in 91.7% (22/24) of these *infants*, among them 50.0% (11/22) of *infants* exhibiting proteinuria that attained nephrotic syndrome (≥ 3+). All of them had reduced serum albumin (ALB) levels, 75.0% (18/24) severely so. Serum cholesterol was determined in 13 *infants*, and hyperlipidemia appeared in only *3/13 of those infants*. Nephrotic syndrome and glomerulonephritis were diagnosed in 45.8% (11/24) and 54.2% (13/24) of the *infants*, respectively. *All the infants with nephrotic syndrome displayed microscopic hematuria and 3 + in urine for occult blood test, confirming to the nephritic-type nephrotic syndrome.* Impaired renal function was reported in 12.5% (3/24) of the *infants*, and these manifested increased levels of serum creatinine level (2/24, 8.3%) or oliguria with elevated blood pressure (1/24, 4.2%).

None of the *infants* had renal tubular dysfunction, since urine-specific gravity, pH values, and urine glucose levels were all within normal limits.

As shown in Table 3, all *infants* tested positive according to the toluidine red unheated serum test (TRUST) and *Treponema pallidum* particle agglutination (TPPA) assay. A positive fluorescent treponemal antibody absorption immunoglobulin M (FTA-ABS-IgM) result was reported in 95.7% (22/23) of children. A majority of the children had increased white blood cell (WBC) counts (75.0%) and C-reactive protein (CRP) levels (91.3%). Other common abnormal laboratory results included decreased hemoglobin (Hb; anemia), increased transaminase, and elevated direct bilirubin levels. X-ray examinations showed periosteal thickening and/or decreased bone mineral density (BMD) *consistent with osseous syphilis*. Compared with children without nephropathy, those with the disease had reduced Hb levels and greater likelihood of anemia, with statistically significant differences (*P* < 0.05).

Sixteen *infants* with CS nephropathy were tested for immune functions. We observed elevated IgM and attenuated C3 in 93.8%% (15/16) and 81.3% (13/16), respectively, and these rates were significantly higher (*P* < 0.05) than the same indices in 48 children without nephropathy.

**Table 2:** Urinalysis and renal-function tests in *infants* with congenital syphilitic nephropathy

	N (total = 24) (cases, percentage, 95% CI of percentage)
*Microscopic* hematuria (microscopic RBCs ≥ 3/HPF) (N, %)	24 (100%, 85.8-100%)
Number of RBCs under microscope (number/HPF), *median (IQR)*	46.7 (6.7–72.6)
*Urine for occult blood test*	
*3+ (N, %)*	*22 (91.7%, 73.0-99.0%)*
*2+ (N, %)*	*2 (8.3%, 1.0-27.0%)*
Proteinuria (N, %)	22 (91.7%, 73.0-99.0%)
Urinary protein ≥ 3+ (N, %)	11/22 (50.0%, 27.3-72.7%)
Urinary protein 2+ (N, %)	8/22 (36.4%, 14.5-58.2%)
Urinary protein 1+ (N, %)	3/22 (13.6%, 2.9-34.9%)
Decreased serum ALB level (N, %)	24 (100%, 85.8-100%)
Mild decrease (30 g/L ≤ ALB < 35 g/L)	2/24 (8.3%, 1.0-27.0%)
Moderate decrease (25 g/L ≤ ALB < 30 g/L)	4/24 (16.7%, 4.7-37.4%)
Severe decrease (ALB < 25 g/L)	18/24 (75.0%, 56.3-93.7%)
Hyperlipidemia (serum cholesterol > 5.7 mmol/L, tested in 13 children) (N, %)	3/13 (23.1%, 5.0-53.8%)
Renal function impairment (N, %)	3 (12.5%, 2.7-32.4%)
Increased sCR △	*2/3 (66.7%, 9.4-99.2%)*
Oliguria, elevated BP	*1/3 (33.3%, 0.8-90.6%)*
Decreased urine-specific gravity (< 1.003) (N)△	0 (0%, 0-14.2%)
Increased urine pH > 7.0 (N)△	0 (0%, 0-14.2%)
Positive urine glucose (N)	0 (0%, 0-14.2%)
Clinical classification of nephropathy (N)	
*Nephritic-type nephrotic syndrome*	*11 (45.8%, 15.8-48.9%)*
Glomerulonephritis	13 (54.2%, 32.7-75.7%)

RBCs = red blood cells; HPF = high-power field; ALB = albumin; sCr = serum creatinine; BP = blood pressure. △Diagnostic criteria for increased sCR refer to previously published literature [23]. Please see Supplementary Materials (S5). Diagnostic criteria for decreased urine-specific gravity and increased urine pH refer to *Pediatrics* [24].

**Table 3:** Comparisons of laboratory test results between children congenital syphilis with and without nephropathy

Laboratory tests	With nephropathy (N = 24) (cases, percentage, 95% CI of percentage)	Without nephropathy (N = 94) (cases, percentage, 95% CI of percentage)
*TRUST titers of dilution, median (IQR)*	*256(80–256)*	*128(112–256)*
TRUST (+) (N, %)	24 (100%, 85.8-100%)	94 (100%, 96.2-100%)
≥ 1:128	*18/24* (75.0%, 56.3-93.7%)	*72/94* (*76.6%*, 67.9-85.3%)
≥ 1:32, < 1:128	*5/24* (20.8%, 3.3-38.4%)	*14/94* (14.9%, 7.6-22.2%)
< 1:32	*1/24* (4.2%, 0.1-21.1%)	*8/94* (8.5%, 2.8-14.3%)
TPPA (+) (N, %)	24 (100%, 85.8-100%)	94 (100%, 96.2-100%)
FTA-ABS-IgM (+) (N, %)	22/23 (95.7%, 78.1-99.9%) (tested in 23 children)	80/93 (86.0%, 78.8-93.2%) (tested in 93 children)
WBC count (×10^9^/L), median (IQR)	18.1 (13.8–22.5)	14.63 (10.32–23.01)
Elevated WBC count (N, %)^a^	18 (75.0%, 56.3-93.7%)	55 (58.5%, 48.4-68.7%)
CRP (mg/L), median (IQR)	39.4 (18.0–86.0)	35.0 (14.5–55.9)
Elevated CRP (> 5 mg/L) (N, %)	21/23 (91.3%, 72.0-98.9%) (tested in 23 children)	84 (89.4%, 83.0-95.7%)
Hb (g/L), median (IQR)*	88.5 (74.7–104.8)	105.5 (85.0–130.3)
Anemia* (N, %)^a^	18 (75.0%, 56.3-93.7%)	47 (50.0%, 39.7-60.3%)
Thrombocytopenia (< 100 × 10^9^/L) (N, %)	7 (29.2%, 9.6-48.8%)	23 (24.5%, 15.6-33.3%)
Increased ALT ^b^or direct bilirubin ^c^(N, %)	12 (50.0%, 28.4-71.6%)	71 (75.5%, 66.7-84.4%)
Abnormal CSF analysis (N, %)^a^	4/23 (17.4%, 5.0-38.8%) (tested in 23 children)	32 (34.0%, 24.3-43.8%)
**Immunology study** ^**b**^	**With nephropathy** **(N = 16)**	**Without nephropathy** **(N = 48)**
IgG (g/L), median (IQR)	8.63 (6.24–12.10)	10.25 (6.82–13.05)
Increased (N, %)	5 (31.3%, 5.7-56.8%)	17 (35.4%, 21.4-49.5%)
Decreased (N)	*0 (0, 0-20.6%)*	*0 (0-7.4%)*
IgA (g/L), median (IQR)	0.38 (0.23–0.47)	0.29 (0.13–0.45)
Increased (N)	*0 (0, 0-20.6%)*	*1 (2.1%, 0.1-11.1%)*
Decreased (N, %)	3 (18.8%, 4.0-45.6%)	20 (41.7%, 27.2-56.1%)
IgM (g/L), median (IQR)*	5.80 (3.58–7.61)	3.14 (1.22–4.24)
Increased (N, %)*	15 (93.8%, 69.8-99.8%)	29 (60.4%, 46.1-74.8%)
Decreased (N)	*0 (0, 0-20.6%)*	*0 (0-7.4%)*
C3 (g/L), median (IQR)*	0.46 (0.37–0.62)	0.71 (0.58–0.87)
Increased (N)	*0 (0, 0-20.6%)*	*0 (0-7.4%)*
Decreased (N, %)*	13 (81.3%, 54.4-96.0%)	18 (37.5%, 23.3-51.7%)
C4 (g/L), median (IQR)	0.07 (0.03–0.13)	0.10 (0.05–0.13)
Increased (N)	*0 (0, 0-20.6%)*	*0 (0-7.4%)*
Decreased (N, %)	9 (56.3%, 28.9-83.6%)	22 (45.8%, 31.2-60.5%)

TRUST = toluidine red unheated serum test; TPPA = Treponema pallidum particle agglutination; FTA-ABS-IgM = fluorescent treponemal antibody absorption immunoglobulin M; WBC = white blood cell; IQR = interquartile range; CRP = C-reactive protein; Hb = hemoglobin; ALT = alanine aminotransferase; CSF = cerebrospinal fluid; IgG, IgA, IgM = immunoglobulins G, A, M. *P < 0.05.

a: Diagnostic criteria for increased WBC count and anemia refer to Pediatrics [24]; Abnormal CSF analysis includes increased WBC count in CSF, increased CSF protein level (diagnostic criteria refer to Pediatrics [24]), or positive CSF TRUST; b: Diagnostic criteria for increased ALT and the normal reference ranges of immunological tests for IgG, IgA, IgM, C3, and C4 refer to the previously published literature [23]; c: Increased direct bilirubin refer to the guideline of the North American Society for Pediatric Gastroenterology, Hepatology, and Nutrition and the European Society for Pediatric Gastroenterology, Hepatology, and Nutrition [25]. Please see Supplementary Materials (S5)

### Treatments and prognosis

Of the 24 *infants* with CS nephropathy, 23 (23/24 [95.8%]) received a 14-day regimen of penicillin G (Supplementary Materials), and 1(1/24 [4.2%]) with impaired renal function dropped out of treatment. Except for one patient who underwent intermittent dialysis due to progressive oliguria and hypertension, the remaining *infants* did not receive additional treatments for nephropathy.

Follow-up information was available for the remaining 21 *infants* except for three *infants* lost to follow-up (these three *infants* included the one who dropped out of treatment). Edema disappeared in all of them, with 89.5% testing negative for proteinuria and 83.3% having no RBCs in their urine. Median durations from the initiation of treatment to the receipt of negative test results were 29.5 and 30.0 days for urinary proteins and RBCs, respectively (Table 4). In 4 *infants* with persistent proteinuria and(or) urinary RBCs, test results were also significantly improved at the last follow-up visits. These *infants* might require longer follow-up time to reach full recovery (S8). Two *infants* with impaired renal function had normal renal-function tests before hospital discharge.

TRUST and TPPA were repeated in 17 *infants*, FTA-ABS-IgM in 15 *infants*. The last test was repeated 1–12 months after treatment. Of all the *infants*, 88.2%*(15/17)* had negative TRUSTs or a titer decrease of ≥ 4 times, while 73.3%*(11/15)* had negative FTA-ABS-IgM results in the repeated tests (Table 4).

**Table 4:** Treatment outcomes of *infants* with congenital syphilitic nephropathy

Symptoms or related laboratory tests	(cases, percentage, 95% CI of percentage)
Disappearance of edema (N, %)	13/13 (100%, 75.3-100%)
Negative proteinuria (3 lost to follow-up)	17/19 (89.5%, 66.9-98.7%)
Time to negative proteinuria (time since treatment initiation, days)	29.5 (10.0–32.3)
Negative hematuria (3 lost to follow-up)	18/21 (*85.7%*, 63.7-97.0%)
Time to negative hematuria (time since treatment initiation, days)	30.0 (17.0–47.5)
Normal renal function (creatinine, hypertension, oliguria) (N, %)	2/2 (100%, 15.8-100%)
Negative TRUST or titer decreased ≥ 4 times (N) (17 *infants* repeated after treatment)	15/17 (88.2%, 63.6-95.8%)
Negative TPPA (N) (17 *infants* repeated after treatment)	0/17 (0%, 0-19.5%)
Negative FTA-ABS-IgM (N, %) (15 *infants* repeated after treatment)	11/15 (73.3%, 44.9-92.2%)

TRUST = toluidine red unheated serum test; TPPA = *Treponema pallidum* particle agglutination; FTA-ABS-IgM = fluorescent treponemal antibody absorption immunoglobulin M

### Comparison between neonates and postneonates

*As shown in*S9, *there was no statistical difference of the incidence of CS nephropathy between neonates and postneonates with early CS, furthermore, no difference was found in urinalysis and renal-function tests between neonates and postneonates with early CS nephropathy.*

*In other respects, compared to postneonates, neonates with early CS were more likely to have Jaundice, while the incidences of hepatosplenomegaly, fever, pale skin,stuffy nose, and cough were higher, and the rates of those with high TRUST titters(≥ 1:128) and elevated WBC count were also higher in postneonates. That the disease seemed to have more signs and symptoms in postneonates might be attributed to the longer interval from symptoms appearing to confirming the diagnosis compared to neonates(Postneonates: median interval from symptoms appearing to confirming the diagnosis: 15 days(IQ25-75: 10–30 days); Neonates: median interval: 3 days(IQ25-75: 0-10days), P < 0.05).*

## Discussion

This study showed that *the prevalence of early CS nephropathy was as high as 19.7% in children with early CS, and this was contrary to previous impressions that this disease was rare.* All of the children with CS nephropathy were young infants < 6 months old. Typical syphilitic skin lesions, hepatosplenomegaly, fever, edema, and GI symptoms were the most common clinical presentations in infants with early CS nephropathy. In terms of laboratory tests, proteinuria was once considered the most prominent feature of syphilitic nephropathy, but in our study we found hematuria in all of the *infants*, proteinuria in 91.7%, diminished C3 levels in 81.3%, and renal function impairment in 12.5% of the patients. Nephritic-type nephrotic syndrome(*45.8%*) and glomerulonephritis*(54.2%)* were diagnosed in these *infants*. *All infants were cured or improved after the syphilis treatment and there was no death or long-term renal damage.*

There are no accurate data on the prevalence of CS nephropathy due to the lack of systematic studies. An earlier study summarized 122 cases of early CS and found no definite cases of syphilitic nephropathy [26]. A recent study in Argentina reviewed 61 cases of CS, including 4 of syphilitic nephropathy, with a prevalence of 6.6%^2^. However, *in this study we found that in children with early CS, the prevalence of syphilitic nephropathy was as high as 19.7%. This difference might be related to age difference, and the mean age of patients enrolled in the former study was 164 days(5.5 months), and the maximum age was 23 months, and in the latter study, the mean age was 2 months, while the maximum age was 8.5 years, so the patients’ age was much older than the present study. In addition, the median titter of TRUST or RPR was not shown in the former study, while in the latter, the median titter of RPR/VDRL is lower than that of TRUST in the present study, so the high titter of TRUST might also be one of the reasons of the high prevalence of CS nephropathy in the present study.* However, we acknowledge that different ethnic groups might possess different prevalence rates of CS nephropathy.

We retrieved a total of 11 articles from PubMed, reporting 25 cases of early CS nephropathy from the 1970s to the present [7, 8, 10−18]. T*hese cases were also all young infants < 6 months old. Among them, 11 cases described the condition of skin involvement, and 5/11(45.5%) of them have typical syphilitic skin lesions, while > 70% of infants in the present study had typical early CS skin lesions. RPR/VDRL titters could be retrieved in 7 of the 11 cases, and the titters in 3 cases(3/7, 42.9%) were ≥ 1:128, while TRUST titters were ≥ 1:128 in 18 of 24(75.0%) in the present study. So we inferred that the high TRUST titter in the majority infants might be the reason for syphilitic skin lesions in the present study.* In addition, we found that hepatosplenomegaly, fever, edema, and GI symptoms were more common in patients with nephropathy than in those without, which might be related to the greater likelihood that *infants* with nephropathy are infected with other pathogens and have GI mucosal edema from hypoproteinemia. The diagnosis of early CS nephropathy is not difficult as long as the clinician is aware of typical early CS symptoms and understands the characteristics of nephropathy.

In addition, a quarter of the *infants* with syphilitic nephropathy in our study did not have typical skin lesions, which could make diagnosis a challenge. Therefore, clinicians must fully understand the features of early CS nephropathy. *Of the 25 previously reported cases of CS nephropathy, 24 had proteinuria, 11 had hematuria, 2 were diagnosed with glomerulonephritis, and the rest 23 were diagnosed with nephrotic syndrome. In 23 infants diagnosed with nephrotic syndrome, 12 had the results about the condition of hematuria or serum levels of complements, and 9 displayed microscopic RBCs ≥ 10/HPF or urine for occult blood test 2+-4+, 4 had decreased levels of C3, C4 and C1q, and 10 of them (10/12, 83.3%)confirmed to nephritic-type nephrotic syndrome.* In the present study, various degrees of hematuria and proteinuria were found in 100% and 91.7% of the *infants*, and nephrotic syndrome and glomerulonephritis were diagnosed in about half and half of them, respectively, and all infants with nephrotic syndrome confirmed to be nephritic-type. Therefore, nephritic-type nephrotic syndrome and glomerulonephritis were the two most common types of early congenital syphilitic nephropathy.

*There was no difference in the incidence of CS nephropathy and in urinalysis and renal-function tests between neonates and postneonates, but the sample was small(there was only five neonates with CS nephropathy), and we could not draw a conclusion from the present study. Compared with neonates, postneonates with CS tended to have more signs and symptoms, and this was in accord with a previous study* [27], *which showed that the incidence of joint swelling with pseudoparesis, skin rash, anemia, snuffles, and periosteal reaction was higher in postneonates than in neonates with early CS, but the study did not show any data about CS nephropathy.*

*Infants with CS nephropathy require syphilis treatment with penicillin, and almost all cases of nephropathy remitted after treatment, with a median time of one month to a negative urine test, with simultaneous reductions in the TRUST titers and a negative conversion of FTA-ABS-IgM. In the previous reported cases, except for two deaths that were not caused by nephropathy, all cases reached remission 1–16 months after treatment, which was consistent with the present study. We posit that satisfactory outcomes can be anticipated after penicillin treatment once an early and correct diagnosis is established.*

Some previous studies have investigated the pathogenesis of CS nephropathy [7, 10, 14, 17]. The typical pathological characteristic was found to be membranous nephropathy, presenting as glomerular mesangial-cell hyperplasia; basement membrane thickening (BMT); and depositions of intraglomerular IgG, IgM, C3, and C4, suggesting that syphilitic nephropathy is an immune complex–mediated injury. All children in the present study had satisfactory prognoses, and no pathological examinations were performed. However, compared with children without nephropathy, those with CS nephropathy had reduced serum C3 levels and elevated IgM levels, suggesting the activation of the alternative complement pathway. This was consistent with the pathogenesis of syphilitic nephropathy suggested by those previous studies.

The strength of our study was that it comprehensively summarized the clinical characteristics, treatments, and prognosis of early CS nephropathy in *infants*. Our results could provide a reference to improve clinicians’ understanding and diagnosis of early CS nephropathy. Similar to other retrospective studies, however, ours had some limitations. There were missing data and patients lost to follow-up. For example, we diagnosed nephrotic syndrome based on qualitative urinary-protein test results alone, since it was difficult to collect urine around the clock for 24-h urinary-protein analysis, which might cause the prevalence of this condition to be underestimated. Previous reported cases also indicated that nephrotic syndrome was more common than glomerulonephritis in this disease (details elaborated in the fourth paragraph of Discussion). Secondly, all children responded well to treatment, so none of them underwent renal biopsy for the pathology study. Because CMV-induced nephropathy could not be ruled out in four children with CMV co-infections, we did not include them in our analysis. In addition, our study was a single-center study. The results might not be applicable to patient populations with different geographical locations and ethnicities.

In conclusion, in this study we showed that CS nephropathy was not rare in *infants* with early CS. Typical CS nephropathy is not difficult to diagnose since the usual symptoms are typical CS skin lesions, hepatosplenomegaly, fever and edema, and laboratory tests usually reflected the nephritic-type nephrotic syndrome or glomerulonephritis. However, a quarter of our *infants* with early CS nephropathy did not have typical syphilitic skin lesions, suggesting that small infants who have nephropathy—especially those with nephritic-type nephrotic syndrome or glomerulonephritis—be screened for CS as an important etiology to avoid misdiagnosis regardless of skin lesions. With prompt diagnosis, the prognosis for *infants* with CS nephropathy is usually satisfactory with standard penicillin G treatment.

## Conclusions

Early CS nephropathy in *infants* often presented with nephritic-type nephrotic syndrome or glomerulonephritis, and the typical skin lesions, hepatosplenomegaly, and edema, etc., were its common clinical presentations. Being familiar with these characteristics could help with the diagnosis. For infants with nephropathy who did not have typical skin lesions, CS should also be screened as an important etiology to avoid misdiagnosis.

### Electronic supplementary material

Below is the link to the electronic supplementary material.

Supplementary Material 1

## Data Availability

The data that support the findings of this study are openly available in Baidu Netdisk at https://pan.baidu.com/s/1IAi69LMQGMiAQQ9coufUCw?pwd=2022, reference number 2022. If it is not available in certain areas, contact the corresponding author and we will provide the data unconditionally.

## List of abbreviations

CS: Congenital syphilis
IQRs: Interquartile ranges
CMV: Cytomegalovirus
GI: Gastrointestinal
RBC: Red blood cell
HPF: High-power field
ALB: Albumin
TRUST: Toluidine red unheated serum test
TPPA: *Treponema pallidum* particle agglutination
FTA-ABS-IgM: Fluorescent treponemal antibody absorption immunoglobulin M
WBC: White blood cell
CRP: C-reactive protein
Hb: Hemoglobin
BMD: Bone mineral density
ALT: Alanine aminotransferase
CSF: Cerebrospinal fluid
