# Supplementary material for: Clinical characteristics of infants hospitalized with early congenital syphilitic nephropathy: a single-center retrospective cross-sectional study in China
Source: BMC Pediatr. 2023 Sep 4;23:442. doi: 10.1186/s12887-023-04250-4 (PMC10476364; doi:10.1186/s12887-023-04250-4)
Supplement: Supplementary file 1 — Supplementary Material 1 [file 12887_2023_4250_MOESM1_ESM.doc]

**Supplementary Materials**

S1. Diagnostic criteria for early congenital syphilis (CS).

Children aged < 2 years meeting one of the following criteria are diagnosed with early CS: (1) dark-field microscopy or silver impregnation staining in tissue samples from skin/mucosal lesions detects *T. pallidum*, or the child is positive for *T. pallidum* nucleic acid; (2) serum is positive for *T. pallidum* IgM antibody; (3) titer of non–*T. pallidum* serological test results at birth is ≥4 times higher than the mother’s titer, and the serological test result for *T. pallidum* is positive; (4) the neonate is born with a negative serological test result for non–*T. pallidum*, or the child’s titer is not four times higher than the mother’s, but the non–*T. pallidum* serological test result turns from negative to positive at the subsequent follow-up examination, or the titer increases with clinical symptoms, and the *T. pallidum* serological test result is positive; and (5) if born to a mother with syphilis, the infant has persistent positive *T. pallidum* serological test results up to 18 months of age.

S2. Diagnostic criteria for congenital syphilitic nephropathy.

Congenital syphilitic nephropathy is diagnosed when the child meets all the following criteria: (1) A definitive diagnosis of CS; (2) evidence of maternal syphilitic infection; (3) evidence of nephritis or nephropathy^*^, but excluding patients with nephritis or nephropathy due to other causes; and (4) disappearance of nephropathic symptoms after adequate syphilis treatment, with no recurrence.

*Evidence of nephritis or nephropathy refers to microscopic examination after urine centrifugal sedimentation shows RBCs ≥ 3/HPF and deformed RBCs >30%, and/or positive proteinuria. Protein compositions include ALB, transferrin, or IgG in the presence of glomerular injury.

S3. Diagnostic criteria for nephrotic syndrome.

(1) Massive proteinuria: 24-h urine protein quantification ≥ 50 mg/kg, or morning urine protein/creatinine (mg/mg) ≥ 2.0, with urinary-protein qualitative test 3+ or 4+ occurring 3× within 1 week; (2) hypoproteinemia: serum ALB < 25 g/L; (3) hyperlipidemia: serum cholesterol > 5.7 mmol/L; and (4) different degrees of edema.

Of the above criteria, (1) and (2) are mandatory for the diagnosis.

S4. Diagnostic criteria for nephritic type nephrotic syndrome

Nephritic-type nephrotic syndrome is diagnosed if the patient with nephrotic syndrome meets one of the following criteria: (1) red blood cells ≥ 10/HFP and confirmed to show glomerulogenic hematuria, and occurring more than three times within two weeks; (2) recurrent or persistent hypertension; (3) renal insufficiency not due to hypovolemia; and (4) persistent hypocomplementemia.

S5. Reference range and abnormal diagnostic criteria for each variable.

1. Increased serum creatinine (sCr):

The level of sCr >49.2μmol/L is considered to be increased in children <1 year old.

1. Increased WBC count:

WBC count >20×10^9^/L is considered to be increased in newborns(0-28 days old), and WBC count >12×10^9^/L is considered to be increased in children between 28 days old and 1 year old.

1. Anemia:

The level of hemoglobin(Hb) <145g/L in newborns, that <90g/L in children between 1 and 4 months old, that <100g/L in children between 4-6 months old, and that <110g/L in children between 6 months old and 5 years old, are considered to have anemia.

1. Increased alanine aminotransferase (ALT):

The level of ALT >45.3U/L is considered to be increased in children <1 year old.

1. Increased direct bilirubin:

The level of direct bilirubin >17μmol/L and > 20% of total bilirubin is considered to be increased.

1. Increased WBC count or protein level in cerebrospinal fluid (CSF):

WBC count in CSF >34×10^6^/L or protein level in CSF >1.2g/L is considered to be increased in newborns; WBC count in CSF >20×10^6^/L or protein level in CSF >0.4g/L is considered to be increased in children <1 year old.

1. Immunological measurements:

The normal range for the immunological index in children <1 year old is as follows: IgG: 3.15-11.41g/L, IgA: 0.23-1.14g/L, IgM: 0.32-1.88g/L, C3: 0.64-1.70g/L, C4: 0.1-0.43g/L.

S6. Treatment options for congenital syphilitic nephropathy.

Intravenous penicillin G: children aged 1–6 days, 50,000 U/kg 2×/day; children aged 7–28 days, 50,000 U/kg 3×/day; children aged >28 days, 50,000 U/kg 4×/day; all the children received 14-day course of treatment.

S7. Comparisons of baseline characteristics between children with congenital syphilis with or without nephropathy

| Characteristics | With nephropathy  (N = 24)  (cases, percentage, 95% CI of percentage) | Without nephropathy  (N = 94)  (cases, percentage, 95% CI of percentage) |
| --- | --- | --- |
| Sex (N, %) |  |  |
| Male | 16 (66.7%, 46.3%-87.0%) | 46 (48.9%, 38.6%-59.2%) |
| Female | 8 (33.3%, 13.0%-53.7%) | 48 (51.1%, 40.8%-61.4%) |
| Age (months, IQR) | 1.6, 1.1–2.4 | 1.5, 0.5–2.5 |
| Minimum | 0.3 | 0 |
| Maximum | 5.8 | 10.2 |
| Premature infants (N, %) | 4/23 (17.4%, 5.0%-38.8%)  (1 abandoned infant, age unknown) | 18/92 (19.6%, 11.3%-27.8%)  (2 abandoned infants, ages unknown) |
| Low–birth weight infants (N, %) | 4 (16.7%, 4.7%-37.4%) | 25/92 (27.2%, 17.9%-36.4%)  (2 abandoned infants, birth weights unknown) |
| Small-for-gestational-age infants (N, %) | 3/23 (13.0%, 2.8%-33.6%)  (1 abandoned infant, gestational age unknown) | 17/92 (18.5%,10.4%-26.6%)  (2 abandoned infants, gestational ages unknown) |
| Maternal history of syphilis (N, %) | 23/23 (100%, 85.2%-100%)  (1abandoned infant, history unknown) | 92/92 (100%, 96.1%-100%)  (2 abandoned infants, history unknown) |

95%CI, 95% confidence limits. IQR, interquartile range.

S8. Pre- and post-treatment comparisons in children with congenital syphilitic nephropathy and persistent proteinuria or hematuria.

| Case number | Pre-treatment | Post-treatment | Follow-up period  (time after treatment, days) |
| --- | --- | --- | --- |
| 1 | Proteinuria (2+) | Proteinuria (1+) | 10 |
| 2 | Proteinuria (3+) | Proteinuria (1+) | 14 |
| 2 | RBCs, 229.5/HPF | RBCs, 33.3/HPF | 14 |
| 3 | RBCs, 44.5/HPF | RBCs, 9.75/HPF | 7 |
| 4 | RBCs, 65.25/HPF | RBCs, 6.15/HPF | 12 |

RBCs = red blood cells; HPF = high-power field.

S9. Comparison between neonates and postneonates

| **Clinical presentations (N, %)** | **Neonates(n=36)** | **Postneonates(n=82)** |
| --- | --- | --- |
| Skin lesions (rash or flaking) | 26(72.2%, 56.9%-87.6%) | 67(81.7%, 73.2%-90.3%) |
| Jaundice* | 18(50.0%, 32.8%-67.2%) | 8(9.6%, 3.2%-16.3%) |
| Hepatosplenomegaly* | 11(30.6%, 14.7%-46.4%) | 51(62.2%, 51.5%-72.9%) |
| Vomiting, bloating, or diarrhea | 10(27.8%,12.4%-43.1%) | 23(28.0%,18.1%-38.0%) |
| Long bone radiographic abnormalities consistent with osseous syphilis | 10/35(28.6%,12.8%-44.3%)  （tested in 35 children） | 34/79(43.0%,31.9%-54.2%)  （tested in 79 children） |
| Fever* | 7(19.4%, 5.9%-33.0%) | 40(48.8%, 37.7%-59.8%) |
| Edema | 5(13.9%, 2.0%-25.8%) | 13(15.9%, 7.8%-23.9%) |
| Pale skin* | 4(11.1%, 0.3%-21.9%) | 25(30.5%, 20.3%-40.7%) |
| Petechiae | 3(8.3%, 1.8%-22.5%) | 4(4.9%, 0.1%-9.6%) |
| Stuffy nose* | 0(0, 0-9.7%) | 27(32.9%, 22.5%-43.3%) |
| Cough* | 0(0, 0-9.7%) | 26(31.7%, 21.4%-42.0%) |
| Pseudoparalysis | 0(0, 0-9.7%) | 7(8.5%, 2.4%-14.7%) |
| Convulsions | 0(0, 0-9.7%) | 3(3.7%, 0.8%-10.3%) |
| **Laboratory tests** | **Neonates(n=36)** | **Postneonates(n=82)** |
| Urinalysis confirmed to CS nephropathy | 5(13.9%, 2.0%-25.8%) | 19(23.2%, 13.8%-32.5%) |
| TRUST* |  |  |
| ≥1:128 | 22(61.1%, 44.4%-77.8%) | 68(82.9%, 74.6%-91.2%) |
| <1:128 | 14(38.9%, 22.2%-55.6%) | 14(17.1%, 8.8%-25.4%) |
| TPPA (+) (N, %) | 36(90.3%-100%) | 82(95.6%-100%) |
| FTA-ABS-IgM (+) (N, %) | 31(86.1%, 74.2%-98.0%) | 71/80(88.8%, 81.7%-95.8%)  （tested in 80 children） |
| WBC count (×10^9^/L), median (IQR) | 17.7（11.9，23.1） | 15.5（11.1，21.4） |
| Elevated WBC count (N, %)* | 13(36.1%, 19.6%-52.6%) | 60(73.2%, 63.4%-83.0%) |
| CRP (mg/L), median (IQR) | 25.2（10.6，56.2） | 38.4（21.2，57.7） |
| Elevated CRP (>5 mg/L) (N, %) | 29(80.6%, 67.0%-94.1%) | 76/81(93.8%, 88.5%-99.2%)  （tested in 81 children） |
| Hb (g/L), median (IQR)* | 134.5（117.5，157.0） | 89.5（80.0，105.0） |
| Anemia (N, %) | 22(61.1%, 44.4%-77.8%) | 43(52.4%, 41.4%-63.5%) |
| Thrombocytopenia (<100×10^9^/L) (N, %) | 13(36.1%, 19.6%-52.6%) | 17(20.7%, 11.8%-29.7%) |
| Increased ALT or direct bilirubin (N, %) | 27(75.0%, 60.1%-89.9%) | 56(68.3%, 58.0%-78.6%) |
| Abnormal CSF analysis (N, %) | 9(25.0%, 10.1%-39.9%) | 27/81(33.3%, 22.8%-43.8%)  （tested in 81 children） |
| **Urinalysis and renal-function tests of infants with nephropathy(N, %)** | **Neonates(n=5)** | **Postneonates(n=19)** |
| Microscopic hematuria (microscopic RBCs ≥ 3/HPF) (N, %) | 5(100%, 47.8%-100%) | 19(100%, 82.4%-100%) |
| Number of RBCs under microscope (number/HPF), median (IQR) | 11.9 (3.5–141.6) | 48.8 (13.5–75.0) |
| Urine for occult blood test |  |  |
| 3+ (N, %) | 4(80.0%, 28.4%-99.5%) | 18(94.7%, 74.0%-99.9%) |
| 2+ (N, %) | 1(20.0%, 0.5%-71.6%) | 1(5.3%, 0.1%-26.0%) |
| Proteinuria (N, %) | 4(80.0%, 28.4%-99.5%) | 18(94.7%, 74.0%-99.9%) |
| Urinary protein ≥3+ (N, %) | 1/4(25.0%, 0.6%-80.6%) | 10/18(55.6%, 30.1%-81.0%) |
| Urinary protein 2+ (N, %) | 3/4(75.0%, 19.4%-99.4%) | 5/18(27.8%, 4.9%-50.7%) |
| Urinary protein 1+ (N, %) | 0(0, 0-60.2%) | 3/18(16.7%, 3.6%-41.4%) |
| Decreased serum ALB level (N, %) | 5(100%, 47.8%-100%) | 19(100%, 82.4%-100%) |
| Mild decrease (30 g/L ≤ ALB < 35 g/L) | 0/5(0, 0-52.2%) | 2/19(10.5%, 1.3%-33.1%) |
| Moderate decrease (25 g/L ≤ ALB < 30 g/L) | 1/5(20.0%, 0.5%-71.6%) | 3/19(15.8%, 3.4%-39.6%) |
| Severe decrease (ALB < 25 g/L) | 4/5(80.0%, 28.4%-99.5%) | 14/19(73.7%, 51.9%-95.5%) |
| Hyperlipidemia (serum cholesterol > 5.7 mmol/L) (N, %) | 0/1(0-97.5%)  (tested in 1 children) | 3/12(25.0%, 5.5%-87.2%)  (tested in 12 children) |
| Renal function impairment (N, %) | 1(20.0%, 0.5%-71.6%) | 2(10.5%, 1.3%-33.1%) |
| Clinical classification of nephropathy (N) |  |  |
| Nephritic-type nephrotic syndrome | 1(20.0%, 0.5%-71.6%) | 10(52.6%, 27.9%-77.4%) |
| Glomerulonephritis | 4(80.0%, 28.4%-99.5%) | 9(47.4%, 22.6%-72.1%) |

**P* < 0.05.
